# Simulation-Based Solutions Reducing Soil and Groundwater Contamination from Fertilizers in Arid and Semi-Arid Regions: Case Study the Eastern Nile Delta, Egypt

**DOI:** 10.3390/ijerph17249373

**Published:** 2020-12-15

**Authors:** Ismail Abd-Elaty, Lorenzo Pugliese, Martina Zelenakova, Peter Mesaros, Abdelaziz El Shinawi

**Affiliations:** 1 Department of Water and Water Structures Engineering, Faculty of Engineering, Zagazig University, Zagazig 44519, Egypt; Eng_abdelaty2006@yahoo.com; 2 Department of Agroecology, Aarhus University, 8830 Tjele, Denmark; Lorenzo.Pugliese@agro.au.dk; 3 Department of Environmental Engineering, Faculty of Civil Engineering, Technical University of Kosice, 04200 Kosice, Slovakia; 4 Department of Construction Technology and Management, Faculty of Civil Engineering, Technical University of Kosice, 04200 Kosice, Slovakia; peter.mesaros@tuke.sk; 5 Environmental Geophysics Lab (ZEGL), Geology Department, Faculty of Science, Zagazig University, Zagazig 44519, Egypt; geoabdelaziz@yahoo.com

**Keywords:** ammonium sulfate application, soil contamination, groundwater contamination, MT3D, numerical modeling, Nile Delta

## Abstract

Intensive agriculture requires increasing application of fertilizers in order to sustain food production. Improper use of these substances in combination with increasing seawater intrusion results in long-term and nonpoint soil and groundwater contamination. In this work, a 3-D groundwater and solute transport numerical model was created to simulate the effect of excessive fertilizers application along the Bahr El Baqar drain system, in the eastern Nile Delta, Egypt. The geotechnical properties of the soils, hydrologic parameters, and unconfined compressive strength were determined at different sites and used as input parameters for the model. Model results showed that silty clay soils are able to contain the contaminations and preserve the groundwater quality. Nevertheless, sandy soils primarily located at the beginning of the Bahr El Baqar drain allow leakage of fertilizers to the groundwater. Thus, fertilizer application should be properly managed in the top sandy layers to protect the groundwater and soil, as increasing aquifer by excess irrigation water increased the groundwater contamination in confined layers due to the high value of cumulative salt for the current situation while the unconfined zone decreased groundwater and soil contamination. A mass transport 3-D multi-species (MT3D) model was set to identify the optimal measure to tackle soil and groundwater contamination along the Bahr El-Baqar drain system. A potential increase of the abstraction rates in the study area has a positive impact in reducing the transfer of fertilizer contamination to groundwater while it has a negative impact for soil contamination. The scenario analysis further indicated that the installation of a drainage network decreases the groundwater and soil contamination. Both solutions are potentially effective for protection against nonpoint contamination along the Bahr El Baqar drain system. However, a more sustainable management approach of fertilizer application is needed to adequately protect the receptors located further downstream in the Nile Delta.

## 1. Introduction

Environmental management of groundwater resources in the Nile Delta is very important due to the increasing water demand and shortage water supply as a result of increasing agriculture, domestic, and industrial consumption [1]. The natural groundwater pollution is caused by geological formations characterized by shallow groundwater mass (water–rock interaction in cold waters), low-quality water infiltration from surface water bodies (streams, rivers, and lakes), seawater intrusion, and geothermal interaction in hot waters. The anthropogenic groundwater pollution is caused instead by improper application of fertilizers and pesticides in agriculture, disposal of industrial and mining wastes, and imperfect well construction [2]. Additionally, coastal areas are suffering from human activities, including over-abstraction, which lead to increased aquifer salinity and groundwater contamination [3,4].

Excessive application of fertilizers produces soil and groundwater contamination through percolation and leaching [5,6]. The degree of leaching depends on a number of factors, including management practices, soil type and corresponding leaching potential, rainfall, and land use [7]. The problem is amplified in regions where groundwater is relatively shallow and open drains are used to remove excess water from intensively or overirrigated soils. The permeability is a key property providing indications for leakage, stability, and settlement of the soil [8]. Void ratio, shape, and grain size are some of the factors affecting soil permeability. According to [9], the physical interaction between fertilizers and soil is predominant in granular soils. Thus, the presence within the soil cap of high-permeable media can facilitate the vertical transport around main drains. However, the permeability of soils may decrease due to the reduction of effective porosity caused by an increase of contaminant concentration [10]. However, the permeability of the contaminated soil with different solutions increased due to the disbanding of soil minerals. The Atterberg limits are deteriorated due to interaction with contaminants [11]. Adaptation of soil physical properties to diverse sources of contamination is a major aspect of more environmental threats accompanied with geotechnical engineering [12,13]. Therefore, the silty clay cover along the southern boundaries of Nile Delta does not offer a complete protection to the shallow aquifers in the area. Integration of ERT with hydrochemical and microbiological data is a guide to understand the surface-groundwater links in order to supply the growing population with safe water [14]. Additionally, incorrect wastewater management and land use distribution lead to severe environmental problems, creating heavy eutrophication condition in surface-water, when surface-water/groundwater relationships exist [15].

Consequently, the geotechnical properties of contaminated soil with fertilizers have not been extensively studied. Several studies have investigated groundwater contamination in the Nile Delta area using different numerical methods. Later studies [16] evaluated the impact of over-abstraction in the eastern Nile Delta showing an increase of groundwater salinity. The authors of [17] developed an integrated model for groundwater contamination from polluted streams using a mass transport three-dimensional multi species (MT3DMS) code. The simulation results suggested the installation of a cut-off wall and the use of linings for polluted drains in order to improve groundwater quality. The authors of [18] carried out numerical study to simulate the efficiency of using different lining materials in protecting groundwater from leakage of open drains. The results showed that geomembrane lining materials provided a better solution for groundwater protection due to the high durability and low cost in comparison with concrete. However, to the best of the authors’ knowledge, there are no modelling studies in the literature simulating groundwater contamination and accounting for the variation of soil properties due to the interaction between fertilizer and soils. The aim of this work was to provide fertilizer management for reducing soil and groundwater contamination in the Eastern Nile Delta. To this end, a numerical model simulating groundwater contamination along the Bahr El Baqar drain system was set up. Four different scenarios were considered to identify optimal simulation-based solutions able to contain groundwater contamination.

## 2. Geologic Setting

The eastern part of the Nile Delta is mostly occupied by the Quaternary and Tertiary deposits. The Quaternary deposits are characterized by the Nile sediments that are occasionally enclosed by wind-blown sands. The sediments constitute variable proportions of sands, clays, and gravels with lateral and vertical variation. Such sediments rest uncomfortably on the older units of Tertiary [19] Pleistocene (old deltaic deposits are classified to Early, Middle and Late Pleistocene). Early Pleistocene deposits are represented by loose sands with cherty pebbles. They increase in thickness northwards with a maximum thickness of about 900 m at El Matariya and decreasing towards the east. Consequently, Middle Pleistocene deposits are known Mit Ghamr Formation. It is represented by sands and gravels with discontinuous occasional clay lenses [20]. The sandy aquifer of the eastern part of Nile Delta is thick with irregular paleo-topography in the form of buried Gizera sands and defunct channels [21]. The upper parts are represented by fluvial sediments.

The depth of these sands in particular and the Mit Ghamr Formation in general represent important parameters in prohibition of pollution of this aquifer. The Late Pleistocene is characterized by intercalations of sands and clays capped by a thin hard crusty sandy limestone bed in some places. Consequently, fluviatile deposits (river sands), fine to medium sands with thin intercalations of clay and silt with a maximum thickness of about 30 m, were hydrogeologically subdivided to clay, which represents the flood plain deposits of the old Damietta Branch. It represents the upper unconfined aquifer with clay intercalations. Its sand/clay ratio reaches up to 50% in the western parts and decreases to the east and north directions. The alternation of coarse and fine sediments within this series supplements the depiction that these sediments are related to the late Pleistocene [22].

## 3. Materials and Methods

### 3.1. Bahr El-Bagar Drain System

**Figure 1:**
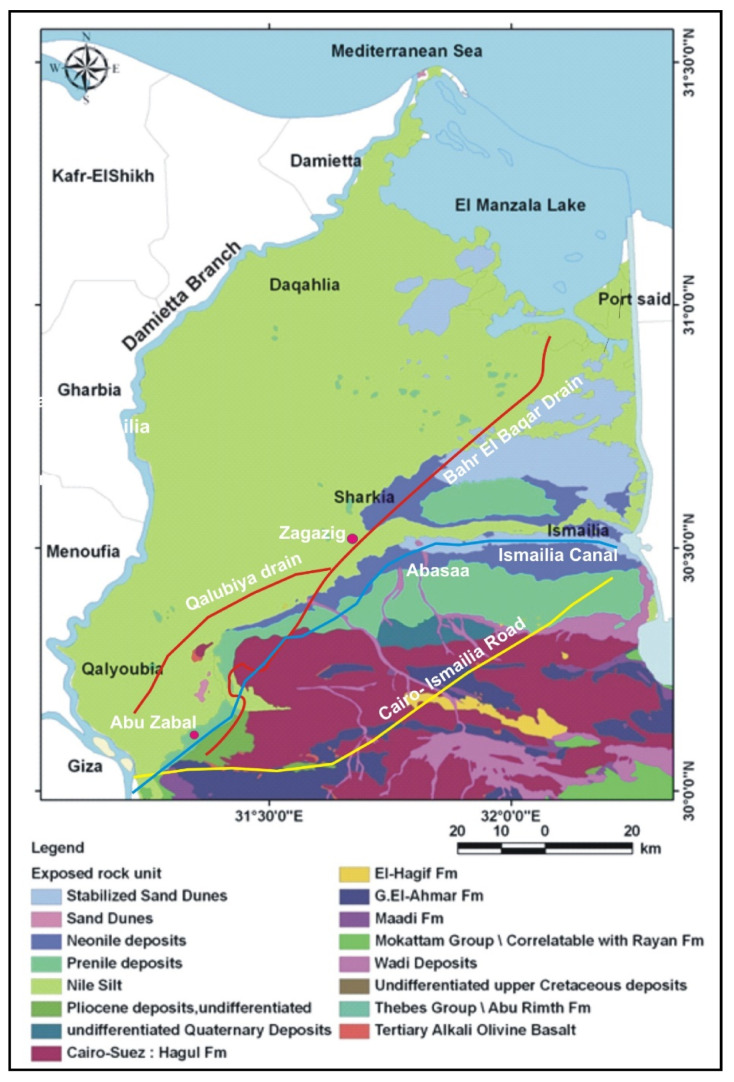
Geologic map of eastern Nile Delta (modified after [21]).

The Bahr El-Baqar drain system consists of two secondary drains (the Bilbeis and the Qalubeya drain), which merge downstream into a main drain (Figure 1). The latter transports water from Zagazig to the southeast sector of the Lake Manzala with an average load of 3 BCM. Agricultural (58%), industrial (2%), and domestic and commercial (40%) sources contribute to the total water flow. The Quaternary aquifer is the main groundwater resource in the study area; it mainly consists of graded sand and gravels intercalated with clay and silt lenses. The aquifer is recharged by infiltration from surface water and direct seepage from Ismailia canal. The lowest contents of such pollutants noticed beside the Ismailia canal reflects the positive hydrochemical impact of this canal on groundwater quality. The study area topography varies from zero level at the coastal line to 18 m at Cairo city above MSL.

### 3.2. Geotechnical Data

**Figure 2:**
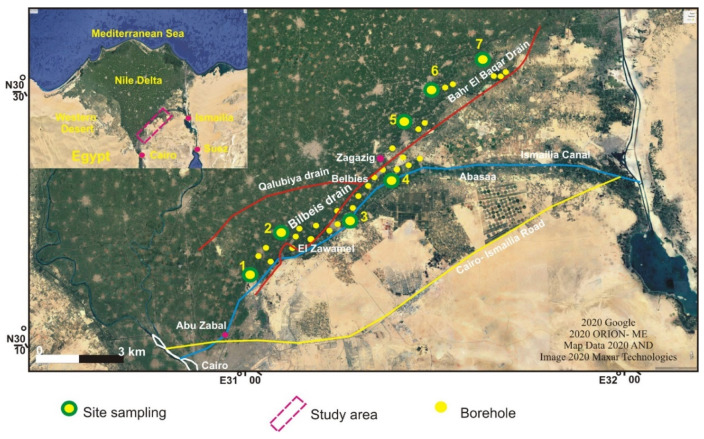
Location map of the study area showing the 7 study sites and the 28 drilled boreholes.

The soil characterization was carried out by field and laboratory tests as an attempt to investigate the relation between contaminant leakage and different geotechnical parameters. Disturbed and undisturbed soil samples were collected from 28 drilling boreholes, at different depths varying up to 10 m. The sampling campaign covered 7 different sites located along the Bahr El Baqar drain system (Figure 2). The soil samples were identified in situ according to a standard practice for description and identification of soils [23]. Additionally, during the drilling boreholes, the in situ standard penetration test (SPT) was also performed, which counts the number of necessary blows to drive the penetrometer 30 cm (N30) down into the soil [24]. Additionally, the uniaxial compressive strength (UCS) test was conducted on undisturbed samples [25].

The laboratory work comprised the evaluation of the Atterberg limits [26], particle size distribution [27], and tests to categorize the soil [28]. Moreover, void ratio and hydraulic conductivity, porosity, and saturation degree were determined for different soil types in all sampling sites [29]. Additionally, the shear strength parameters (friction angle and cohesion) were determined; hence, soil samples were filled in the 10 × 10 × 2 cm space in five layers to form a controlled density. Finally, the results were investigated to advise the effect of different contamination sources on the geotechnical parameters.

Chemical analysis for 42 soil–water extracts was carried out including total dissolved salts (TDS), pH, chloride ion (Cl^−^) and sulfate ion (SO_4_^2−^) and nitrates (NO^3−^) concentrations. Samples were analyzed using the standard analytical procedures [30] and British Standard [31]. All of the hydraulic parameters, such as void ratio (e), hydraulic conductivity (K), porosity (n), and the nitrates pollutant as fertilizer concentration (c), were collected and used as an input parameters for numerical modeling as presented in Equations (1) and (2), while the other parameters for specific yield (Sy), longitudinal dispersity (α_L_), the lateral dispersity (α_T_), and the vertical dispersity (α_V_) were used based on the previous studies for the current study area.

### 3.3. Numerical Model

A three-dimensional groundwater flow and solute transport model was created using VISUAL MODFLOW 2000 [32]. The model combines a MODFLOW and a MT3D code.

The differential equation for groundwater flow used in MODFLOW is the following [33]:

(1) $$\frac{\partial }{\partial x}\left(K_{xx}\frac{\partial h}{\partial x}\right)+\frac{\partial }{\partial y}\left(K_{yy}\frac{\partial h}{\partial y}\right)+\frac{\partial }{\partial z}\left(K_{zz}\frac{\partial h}{\partial z}\right)+q=S_{s}\times \frac{\partial h}{\partial t}$$

**Figure 3:**
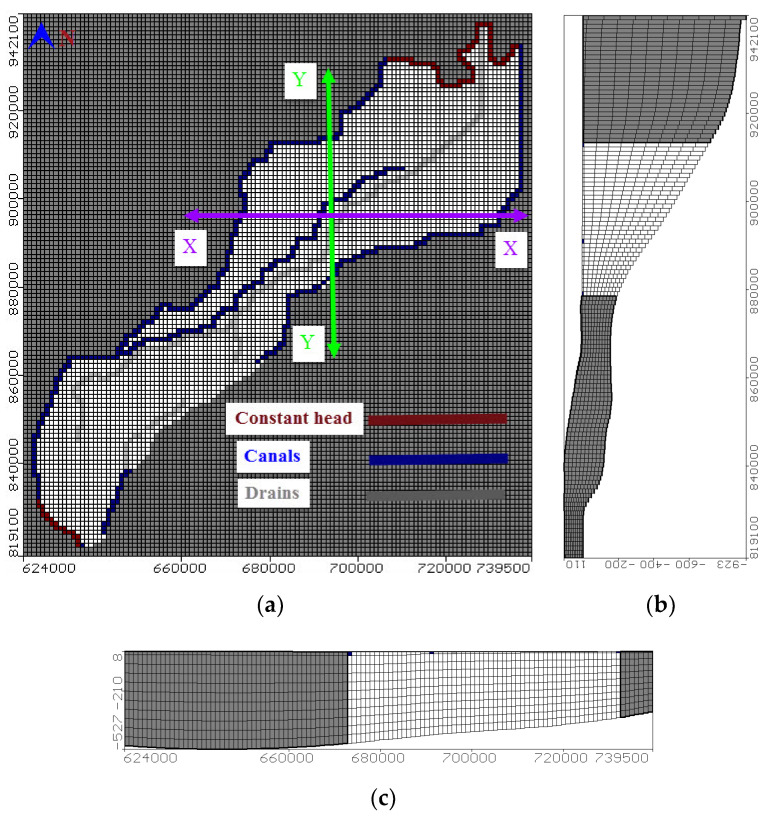
Numerical model geometry and boundary condition (**a**) a real view, (**b**) X-direction from East to West and (**c**) Y-direction from North to South.

The differential equation for the solute transport model of MT3D is the following [33]:

(2) $$\frac{\partial C}{\partial t}=\frac{\partial }{\partial x_{i}}\left(D_{ij}\frac{\partial c}{\partial x_{j}}\right)+\frac{\partial }{\partial x_{i}}\left(v_{i}c\right)+\frac{q_{s}}{\theta }\left(c_{s}\right)+\sum_{k=1}^{N}R_{k}$$

where *Kxx*, *Kyy*, and *Kzz*: aquifer conductivity (LT^−1^), *h*: flow head (L), *q*: sources and/or sink of water (T^−1^), *Ss*: specific storage (m^−1^), *t*: time (T), *C*: groundwater concentration (ML^−3^), *Dij*: dispersion coefficient (L2T^−1^), *V*: seepage velocity (LT^−1^), *qs*: water flux of sources (positive) and sinks (negative) (T^−1^), *Cs*: sources or sinks concentration (ML^−3^), *n*: media porosity [dimensionless] and *Rk* is the chemical reaction term (ML^−3^T^−1^). The study area was defined by 124 (row) × 116 (column) for active and inactive cells with square cell area of 1 km^2^ (Figure 3a). The model domain was divided into 10 layers. The top layer represented a clay cap having a varying depth of 20 m (south) to 50 m (north). This cap defined the Quaternary aquifer as a semi-confined aquifer. Additional layers presented an equal thickness, which increased moving from the south (200 m near Cairo) to the north (1000 m at coastal line). Figure 3b,c presents the vertical model sections in the y direction from the north to east and in the X-direction from the east to west, respectively.

### 3.4. Model Boundary Conditions, Recharge, Abstraction, and Hydraulic Parameters

A constant head of zero above mean sea level (M.S.L.) was assigned along the Mediterranean shore line at the north while a head of 16.50 m at south part to represent the river Nile. The East boundary was the used river package and started 16.15 m in the south to 0.50 m in the north while the west is started by 14.50 to 0.50 m above MSL in the north as presented in Figure 3a. The drains’ heads were assigned using a drain package and ranged from 12 m in the south to 0.25 m in the north.

**Figure 4:**
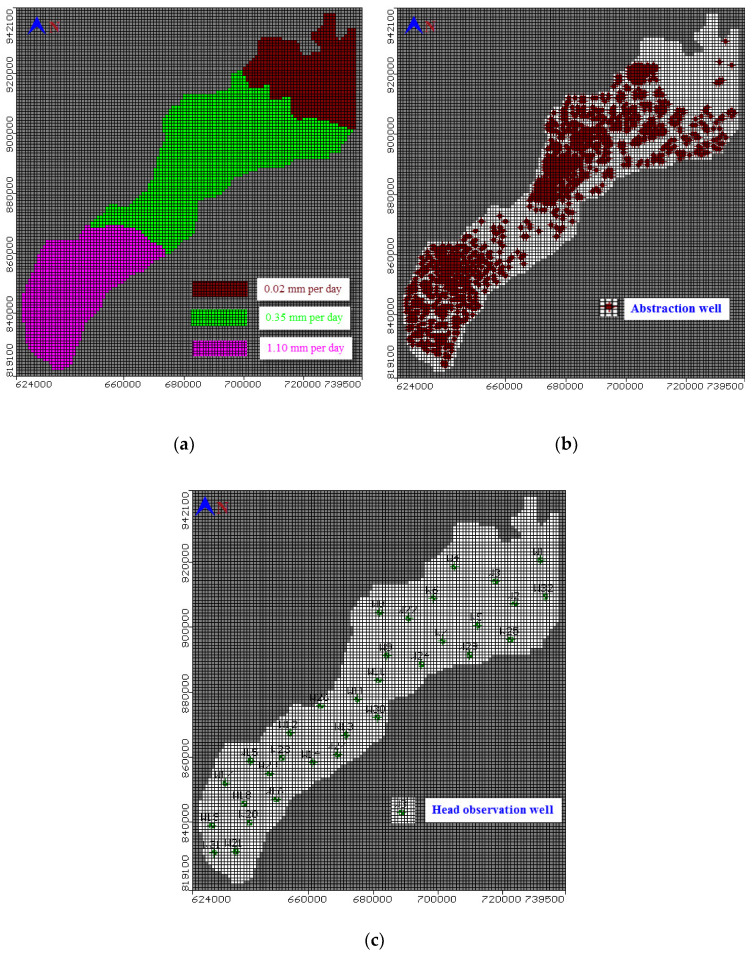
Model (**a**) net recharge, (**b**) abstraction wells, and (**c**) observation head wells.

The groundwater in the study area is recharged by rainfall with an average precipitation of 25 mm/year, downward leakage due to excess irrigation water, and canals infiltration towards the aquifer, which ranges between 0.05 and 1.10 mm/day, inter-aquifer flow to groundwater [34]. Recharge contribution by the different processes is uneven. Thus, a number of three different recharge regions were considered (Figure 4a). The study area presents two types of production wells: governmental wells for irrigation and drinking water wells (Figure 3b). The abstraction data for 1992, 1995, 1997, and 2002 were taken from the RIGW inventory and provided to the model. Abstraction rates showed a sharp increase over the last 30 years, which reached 7.00 BCM in 2016 [35]. The total abstraction rates from the current study reached 1,702,000 m^3^ per day. Nitrate in the studied groundwater samples ranges between 1 to 25 mg/L with an average of about 12 mg/L [36]. The current simulation is carried out by studding the effect of nitrates concentration (NO^3−^), which represented the fertilizers’ effect on groundwater. The contaminant concentration was simulated by assigning a recharge concentration of 3.60, 6.94, and 8.90 ppm [37] for the recharge region having the lowest (0.02 mm/day), medium (0.35 mm/day), and highest net recharge (1.10 mm/day), respectively. The distribution of the observation well for monitoring the groundwater head is shown in Figure 4c. The hydraulic parameters for the top clay layers, which are used in the current model for hydraulic conductivity and porosity, are presented in Table 1 while the specific yield is 0.15 m^−1^. The quaternary aquifer values for the hydraulic parameters used in the current model are the horizontal hydraulic conductivity, which ranged from 5 to 100 m/day and increased by depth; the specific storage (S_s_) ranged from 0.005 to 0.0005 m^−1^, specific yield (S_y_) ranged from 0.15 to 0.20’ and total porosity ranged from 30% to 20% [1]. The longitudinal dispersivity (α_L_), the lateral dispersivity (α_T_), and the vertical dispersivity (α_V_) were set equal to 250, 25, and 2.50 m, respectively. The diffusion coefficient was set equal to 10^−4^ m^2^/day [38].

The calibrated model was used to simulate four scenarios of groundwater contamination produced by extensive use of fertilizers (scenario 1), over pumping (scenario 2), recharge by rice cultivation (scenario 3), and increased drainage network discharge (scenario 4). For scenario 1, the fertilizer concentration was increased following four consecutive steps. The first step increased the concentrations by 20% to reach 4.32, 8.33, and 10.68 ppm; the second by 40% to reach 5.04, 9.72, and 12.46; the third by 60% to reach 5.76, 10.10, and 14.24 ppm; and the fourth by 80% to reach 6.48, 12.49, and 16.02 ppm. Increasing the concentration of fertilizers is due to the expected use of these elements in the future, which is a result of the extensive use in agriculture for balancing the increase in population and to increase crop productivity. Moreover, in Egypt, the land is cultivated by three seasons to overcome population growth and save food security. For scenario 2, the abstraction rates were increased by 25, 50, 75, and 100%. Scenario 3 considered the increasing natural recharge due to the use of rice cultivation following four consecutive steps in the three different recharge regions. The first step increased the natural recharge by 20% to reach 8.76, 153, and 481.8 mm/year; the second by 30% to reach 9.49, 165.75, and 521.95; the third by 40% to reach 10.22, 178.50, and 562.10; and the fourth by 50% to reach 10.95, 191.25, and 602.25 mm/year. For scenario 4, the drainage network discharge was increased by 25%, 31%, 41%, and 69% in comparison with the base case to reach 760,650, 793,000, 858,400, and 1,023,400 m^3^/day.

## 4. Results and Discussion

### 4.1. Geotechnical Investigation

**Figure 5:**
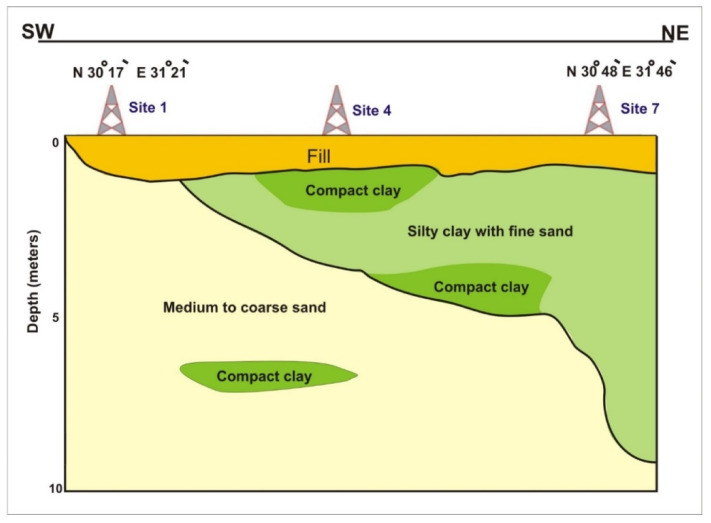
Geological cross section showing the soil profile along the study sited based on drilled boreholes at sites 1, 4, and 7 (for location, see Figure 1).

The soils were composed of silty clay with fine sand, compact clays with sand intercalations [28], and topped with a 1-m fill layer (Figure 5). Silty clay with fine sand (MH) was located near the surface and reached a depth of 9 m at site 7 towards the end of the Bahr El-Baqar drain. The narrow particle size distribution of MH prevented a reduction of the volume of voids, favoring the transport of contaminations. It contained >12% fines with compact clay lenses in some places, which was underlain by medium to coarse sand (SM) especially in the beginning of Bahr El-Baqar drain. However, these uniform sand soils do have an important use as drainage materials. The relatively large and permanent void spaces acted as conduits to carry water [39]. Furthermore, the water content (Wc), liquid limit (WL), and plastic limit (WP) were highest at site 6 and 7, lower at site 4 and 5, and lowest at site 2 and 3 (Table 1) as a result of organic contaminants’ presence [40,41]. Therefore, the soil heterogeneity was the main factor controlling leaching from the encased ponds and open drains.

#### 4.1.1. The Hydraulic Conductivity and Porosity

Values of *K* and *n* varied across the different study sites. In the beginning of Bahr El-Baqar drain (Khanka), *K* was equal to 0.136 m/day and n reached 55.88% and 62.42% for the sand and silty clay, respectively (Table 1). Along Belbies drain (El Zawamel), the *K* value decreased to 0.124 m/day and porosity increased to 68.75% and 78% for the sand and silty clay soil, respectively. Between Ismailia Canal and Belbies drain, *K* increased to 0.140 m/day and n decreased to 55.88% and 70.96% for the sand and silty clay soil, respectively. In the northern part of the study area, the average *K* was two orders of magnitude lower in comparison to the previous study sites (0.0023 m/day). The reduction of *K* and *n* value at site 6 and 7 is due to the obstruction of the pores generated from the chemical reaction between suspended solids and microorganisms. Values of n were also the lowest and equal to 40.43% and 52.63% for the silty sand and silty clay soils, respectively. The lower *K* value at site 6 and 7 indicates that these soils play an important role as a cap extent until a depth of 5.5 m. This prevents most of the fertilizers reaching the groundwater and restricts the effect of these contaminants on the surface water and groundwater level during the drilling borehole range between 1.8 and 2.9 m.

#### 4.1.2. The Mechanical Properties

The SPT values of sand soil at sites 1–5 ranged from 28 to 32 and for silty clay with fine sand soil varied from 7 to 12, which is classified as medium-dense sand and medium stiff clay. The SPT values for silty clay with fine sand soils are 50 and 14 at site 6 and 7, and classified as dense soils (Table 1).

The friction angle (φ) ranges from 25° to 30° in sand soils with high relative density ranging from 70–85% and ranges from 7° to 12° for silty clay with relative density from 20 to 40% in the same direction along Belbies drain. In sites 1, 2, 3, 4, and 5 along Belbies drain (Figure 1), it ranged from 1.1 to 1.45 kg/cm^2^ due to facilitated sliding of soil particles, such as the lubricating effect, while the contaminants occupied the pore spaces. While UCS increased in the sites (6 and 7) at the end of Bahr El Baqar drain, it reached 1.52 kg/cm^2^ as the percentages of contamination decreased. The results revealed that the Atterberg limits were reduced by increasing the fertilizer percentages with the recharge rates (Figure 4a) and over pumping (Figure 4b), which causes the silty clay soil to behave more like a granular material, leading to a decrease in viscosity and causing low resistance. Accordingly, the porosity and hydraulic conductivity of soil contaminated with fertilizers, based on bulk and dry weight, were decreased with the increase of fertilizer percentages that occupy the pores inside the soil particles. Additionally, the results of the SPT values and the degree of friction angle modification depends on the concentration of the fertilizers and the soil type, where the reduction of SPT values and friction angle degrees significantly increased the volumetric strains of sand soils, which can be attributed to coating the sand soil particles with fertilizers. Consequently, the UCS of silty clay soil considerably slightly reduced as the percentages of fertilizers increased.

#### 4.1.3. Chemical Analysis for Soil-Water Extracts

The chemical analysis of soil water extracts showed that the chloride (Cl^−^) and sulfate (SO_4_^2−^) and nitrates (NO^3−^) concentration were increased from site 1 to site 7 along the Bahr El-Baqar drain. Consequently, the chloride (Cl^−^) and sulfate (SO_4_^2−^) and nitrates (NO^3−^) concentrations in soil water extracts in the beginning of Bahr El-Baqar drain (site 1) are 270, 170, and 16 mg/L, respectively, and in the ending of Bahr El-Baqar drain (Site 7) 390, 270, and 46, respectively (Table 1). Hence, the thickness of sand increases in the beginning of Bahr El-Baqar drain and it reaches 10 m, thus playing an important role as the drainage zone favoring the leaching of contaminant diffusion. While, in the ending of the Bahr El-Baqar drain, the soil water extracts (at the northern part), and the diffusion of contaminants is restricted and it is clear in the increase of the chloride ion (Cl^−^) and sulfate ion (SO_4_^2−^) and nitrates (NO^3−^) concentrations (Table 1) than in the beginning of the Bahr El-Baqar drain (at the southern parts). As well as in other recent studies at the same sites, these concentrations in the groundwater wells in the beginning of Bahr El Baqar drain at depths (18–40 m) are higher than the ending parts of Bahr El Baqar drain depending on the absence of the silty clay cap [42]. In general, the water from the drain seeps into the permeable layers and is finally pumped up again. Otherwise, Ismailia canal is the main source of recharge for the Quaternary aquifer in the study area through the open drain, which leads to a decrease in the concentration of contamination in accordance with the void ratio, high porosity, and hydraulic conductivity. Seepage from the canal is estimated to provide about 3,500,000 m^3^/day of recharge whereas the combined input from irrigation water and rainfall provides about 1,600,000 m^3^/day of recharge to the aquifer [42,43].

### 4.2. Hydrological Investigation

**Figure 6:**
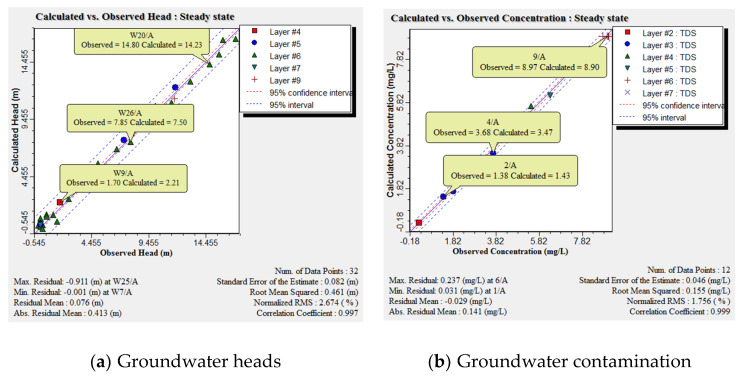
Observed groundwater results versus calculated ones.

**Figure 7:**
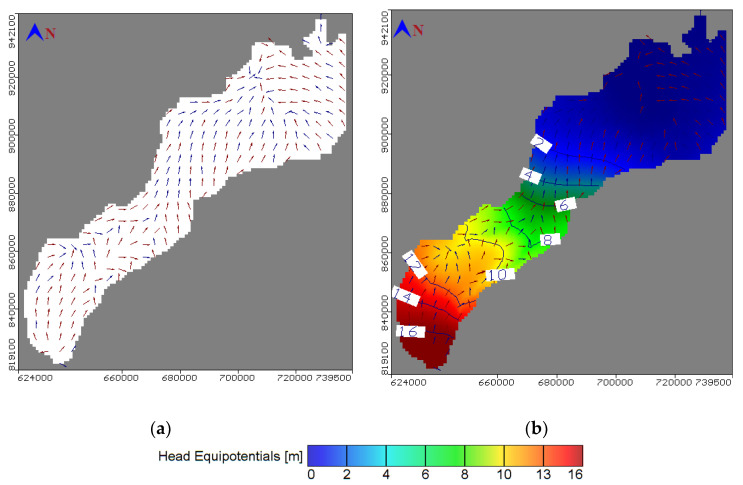
Aerial view of groundwater for (**a**) velocity distribution and (**b**) calculated heads.

**Figure 8:**
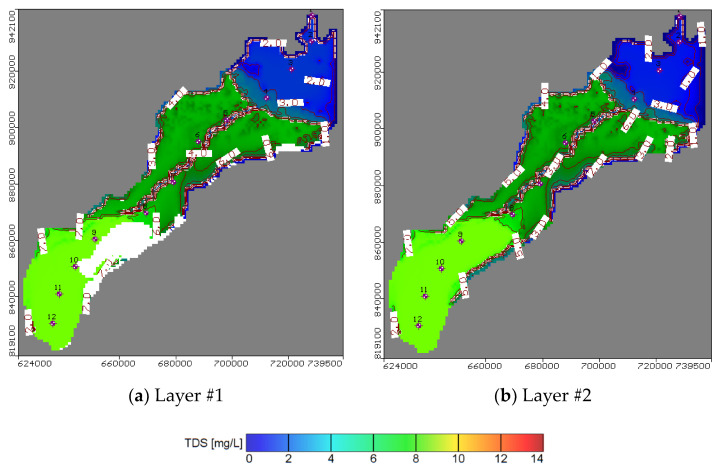
Aerial view of groundwater contamination of Nitrates fertilizer in NDA.

The calibration procedure involved 32 observation wells, which were homogenously distributed along the study area. The differences between the piezometric heads measured in the observation well and the calculated heads simulated by the model are presented (Figure 6a). The residual piezometric head ranged between 0.058 and 1.129 m with a root mean square error (RMSE) of 0.542 m, residual mean of 0.168 m, absolute mean residual of 0.47 m, and normalized root mean square of 3.052%. The groundwater velocity and head is presented in Figure 7a,b respectively from high head at the south near Cairo to low at the north connected by Mediterranean Sea. The results of the salt transport model showed a residual concentration ranging between 0.031 and 0.237 ppm (Figure 6b). The root mean square (RMS) reached 0.155 m with a normalized root mean square of 1.756%, thus proving satisfactory model results. Moreover, fertilizer contamination resulting from agriculture activities is presented in (Figure 8a) for the top clay cap layer and (Figure 8b) for the quaternary first layer. The result showed that the area is covered by a silty clay cap layer, which is able to contain the contamination. Additionally, the covering soils extending to a depth of 5.5 m play a control role and prevent most of the contaminations from reaching the groundwater. The effect of the fertilizer contamination is thus restricted to the superficial water characterized by a water level ranging between 1.8 and 2.9 m. The area without clay cap is subjected to high fertilizer concentration values while the thickness of silty sand soils increased in the beginning of Bahr El Baqar drain and played an important role as the drainage zone, which allows the most contamination to reach the groundwater. The results indicated that the contamination is high in the south due to the high values of aquifer recharge to groundwater while it decreases in the north due to the low values of recharge. The model further indicated that the fertilizer present in clay soil generates low risk to groundwater contamination and high risk to soil. Otherwise, in the sand soil, the fertilizer accumulation has high risk for groundwater contamination and low risk for sand soil.

### 4.3. Future Scenarios of Groundwater Fertilizer Contamination

**Figure 9:**
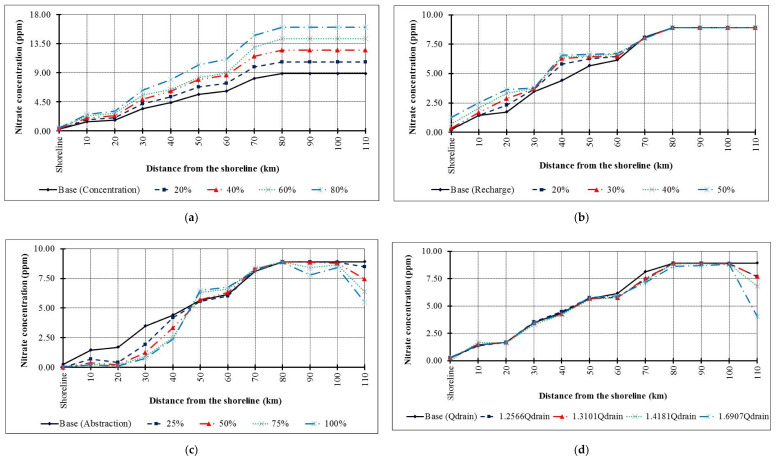
Simulated fertilizer concentration (ppm) at different distances from the shoreline for (**a**) extensive use of fertilizer (scenario 1), (**b**) recharge by rice cultivation (scenario 2), (**c**) over pumping (scenario 3), and (**d**) increased drainage network discharge (scenario 4).

**Figure 10:**
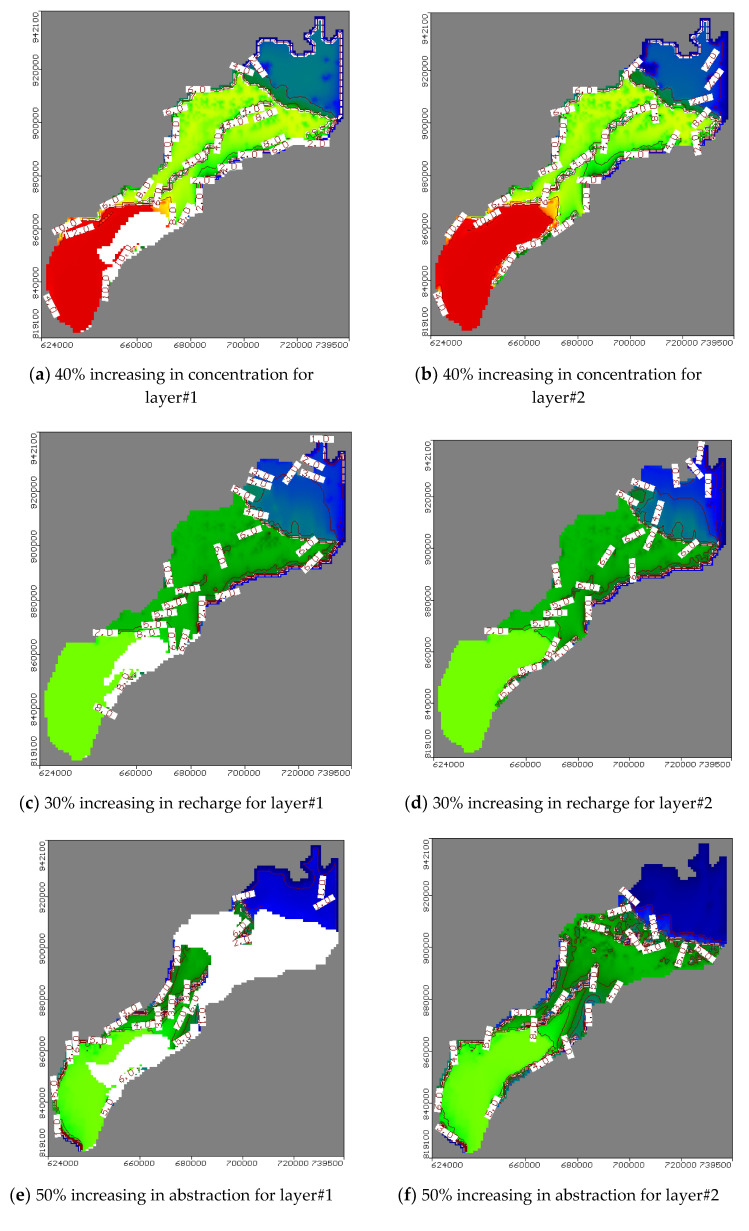

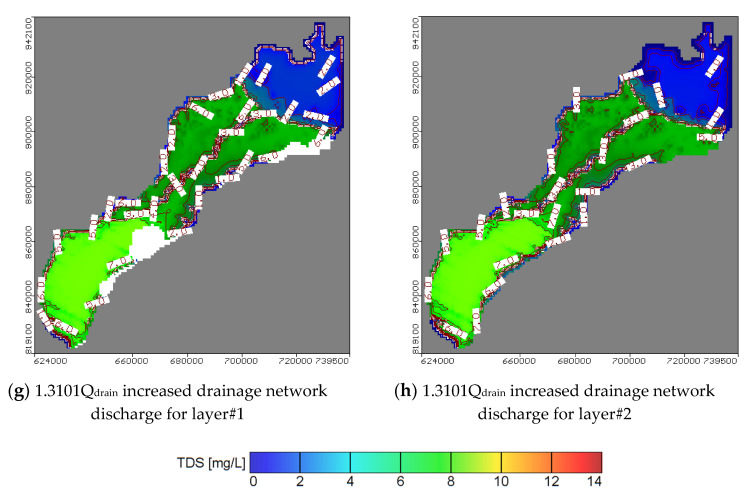
Aerial view of groundwater contamination of nitrates fertilizer in NDA.

The model results for scenario 1 indicated that with an extensive use of fertilizers, the groundwater is contaminated (Figure 9a and Figure 10a) and the high value occurred in the lower part at site 1 and 7 more than the upper part at site 5, 6, and 7 due to the clay cap. The soil contamination in the top layer was decreased at site 1 and 2 in the lower part (sand zones) due to transfer of the fertilizers to groundwater compared with the middle part at site 3 and 4 and upper part at site 5, 6, and 7 (clay zones) as shown in Figure 10b. Thus, the applied fertilizers should be managed in the top sand areas to protect the freshwater body.

The model results for scenario 2 indicated that by increasing the aquifer recharge due to rice cultivation, the aquifer contamination is also increased in confined layers while it is decreased with low changes in the unconfined zone (Figure 9b and Figure 10c).

In particular, the upper and the central part of the study area are subject to more contamination due to the confinement of these zones due to the high value of cumulative salt for the current situation, which is transferred by high recharge rates (Figure 9b and Figure 10c). Additionally, this part is connected by the Mediterranean Sea in the north and is affected by high salinity due to saltwater intrusion and all drinking water is turned off. The lower part is managed from fertilizer contamination due to high recharge and an increase of the aquifer capacity. The top soil layer is contaminated and decreases gradually from clay zone at site 1 and 2 to sand zone at sites 5, 6, and 7, which have high recharge rates (Figure 10d). Thus, fertilizer application must be reduced in the lower part to safe groundwater while the upper part is used for the desalination and irrigation wells.

The model results for scenario 3 indicated that over pumping reduced the groundwater contamination (Figure 9c and Figure 10e). This may be explained by the distribution of abstraction wells (Figure 4b and Figure 10e). In fact, the upper and lower parts are subjected to high abstraction compared with the central with low abstraction and low high contamination. Additionally, the results showed that with increasing abstraction rates, the groundwater head is lowered in clay layers more than in sand layers. The head decrease minimizes leaching towards the groundwater. This indicates that over abstraction has a positive impact on the transfer of the fertilizer contamination to the groundwater while it has a negative impact to increase the contamination in the top soil layer with zones of over pumping at site 1 and 2 in the lower part and 5, 6, and 7 in the upper part, but it decreased in central part at site 3 and 4 for low abstraction rates (Figure 10f).

The model results for scenario 4 indicated that the fertilizer contamination may be decreased by increasing the discharge of the drainage system network (Figure 9d and Figure 10g). Moreover, the effect is amplified in the lower part of the study site due to the absence of a clay cap, which would otherwise decrease the drainage discharge. Moreover, the salinity of the top soil layer is decreased by increasing the discharge of this horizontal drainage system for the lower part at site 1 and 2 (sand zone) and decrease gradually in the north direction for the central and upper part due to the high thickness of the clay cap for the confined layer and low values of drainage discharge (Figure 10h).

## 5. Conclusions

Extensive application of fertilizers in the eastern Nile Delta produces diffuse pollution and has a negative effect on soil and groundwater conservation. A mass transport 3-D multi-species (MT3D) model was set in this work to identify the optimal measure to tackle soil and groundwater contamination along the Bahr El-Baqar drain system.

The adaptation of geotechnical properties of contaminated soils is mainly according to the interaction between contaminant and the soil particles. The porosity and hydraulic conductivity of contaminated soils decreased due to the fertilizers occupying the pores between the soil particles. Furthermore, Atterberg limits, porosity, hydraulic conductivity, and UCS are reduced with excessive fertilizer supplementation. Consequently, the UCS of silty clay soil considerably slightly reduced as the percentages of fertilizers increased due to a lubricating effect, which facilitated sliding soil particles. Otherwise, the SPT and friction angle decreased with an increase of the volumetric strains of sand soils, which can be attributed to coating the sand soil particles with fertilizers.

The model results indicated pronounced soil heterogeneity in the study area, which is a key factor controlling the leaching velocity from the uncased ponds and open drains. In the northern part, the silty clay layer acts as a cap and hinders the contamination, preventing most of it from reaching the groundwater. Further downstream, in the beginning of Bahr El-Baqar drain, the silty sand soils function as a drainage zone, which allows the contamination to reach the groundwater. The scenario analysis further indicated that an increase in fertilizer use or a higher recharge would increase the aquifer contamination in confined zones while the groundwater and soil contamination were decreased in the unconfined zone due to high rates of recharge. On the contrary, over pumping may produce a decrease in the groundwater contamination due to a decrease in the groundwater head and the minimization of connection between the groundwater and the fertilizer contamination. Additionally, the installation of a subsurface drainage system may decrease the groundwater head and manage the contamination to transfer in groundwater. Accordingly, these two last solutions are recommended to minimize soil and groundwater contamination in the investigated area. The consequences significantly support minimization of the connection between the soil and groundwater with fertilizer contamination. Consequently, the contamination and the level of protection must be more carefully determined, where the fertilizers rapidly downward flow into the soil and shallow sand aquifer due intensive pumping from the vadose zone. Additionally, the fixing of a subsurface drainage system decreased the groundwater head and managed the contamination to transfer in groundwater.

However, additional investigation is required to consider the impact of excessive fertilizers on the geotechnical properties of the different types of soils, especially compressibility and compaction characteristics.

## Figures and Tables

**Table 1:** Soil properties and geochemical parameters at the different study sites.

Site	1		2 and 3		4 and 5		6 and 7	
Soil type	Sand	Silty clay	Sand	Silty clay	Sand	Silty clay	Silty clay	Silty clay
Porosity, *n* (%)	55.88	62.42	68.75	78	55.88	70.96	40.43	52.63
Bulk density, (g/cm^3^)	1.74	1.78	1.78	1.71	1.74	1.82	1.84	1.75
Dry density, (g/cm^3^)	1.70	1.65	1.68	1.51	1.70	1.55	1.52	1.45
Specific gravity	2.65	2.68	2.73	2.7	2.65	2.65	2.73	2.71
Wc, (%)	-	14	-	14	-	23	-	27
WL, (%)	-	35	-	54	-	75	-	80
WP, (%)	-	22	-	28	-	30	-	33
Hydraulic conductivity, k (m/day)	0.136		0.124		0.140		0.002	
SPT	32	12	28	7	32	12	50	14
UCS. (kg/cm^2^)	-	1.1	-	1.22	-	1.45	-	1.52
Friction angle, φ (°)	30	-	25	-	25	12	-	7
TDS. (mg/L)	600		1200		2680		3600	
Nitrates, (mg/L) for soil water extracts	16		22		36		46	
Nitrates, (mg/L) for groundwater	10		7		5		3	
Chloride, (mg/L) soil water extracts	270		320		350		390	
Sulphate, (mg/L) soil water extracts	170		200		250		270	
